# Study on the effect of acupunture treatment on autonomic nerve dysfunction in convalescent period of stroke based on heart rate variability assessment technique

**DOI:** 10.1097/MD.0000000000032355

**Published:** 2022-12-16

**Authors:** Shijing Jia, Wang Lu, Minghui Hang, Chu Zhang, Zilin Ma, Kun Xue, Yunqi Lu, Shenghong Zhang, Yijin Guo, Jiabao Zhang, Xinyu Zhang, Yimeng Wang, Haiyin Zhao

**Affiliations:** a Longhua Hospital, Shanghai University of Traditional Chinese Medicine, Shanghai, China; b Shanghai University of Traditional Chinese Medicine, Shanghai, China; c Shanghai Changning Tianshan Traditional Chinese Medicine Hospital, Shanghai, China; d Shanghai Fifth People’s Hospital, Shanghai, China.

**Keywords:** autonomic dysfunction, clinical trials, heart rate variability, stroke

## Abstract

Stroke patients with autonomic dysfunction are more likely to develop cardiac problems, which have been linked to lower functional outcomes and increased mortality. In this study, heart rate variability (HRV) detection paired with the Clinical Feature Scale will be utilized to elucidate the immediate impact of manual acupuncture on autonomic dysfunction of varying severity in the convalescence stroke phase.

This is a randomized, single-blind, controlled clinical trial approach. At a ratio of 1:1, 60 appropriate patients will be randomly randomized into either the experimental or control group. On the basis of symptomatic treatment drugs, the experimental group will additionally undertake acupuncture therapy 3 times a week for 4 weeks, for a total of 12 times. Primary outcomes include 24-hour HRV and 60-minute HRV detection at week 4 compared with baseline. The secondary outcome is the score of clinical feature scale at week 4 compared with the baseline. Adverse events and safety indices will be recorded throughout the experiment. The SPSS V.25.0 statistical program was applied for analysis, and measurement data were expressed as mean ± SD.

## 1. Introduction

Stroke may be followed by central autonomic dysfunction,^[1,2]^ with an early occurrence of from 21.8% to 79%,^[3–5]^ including cardiovascular, endocrine, inflammatory immune, digestive, urinary and other systems.^[6,7]^ The clinical signs include brain-heart syndrome, stress hyperglycemia, shoulder-hand syndrome and sweating problem, and severe instances may lead to sudden cardiac death.^[8]^ Therefore, early detection and treatment of post-stroke autonomic dysfunction cannot be neglected and has major prognostic relevance.

At present, contemporary medical treatment techniques for post-stroke autonomic dysfunction include symptomatic drugs, but difficulties such as no agreement on the dosage,^[9]^ noable side effects^[9–11]^ and complications^[12]^ exist. Therefore, it is vital to investigate a safe, effective, easy, and inexpensive therapy. Acupuncture is commonly employed in traditional Chinese medicine therapies. Studies^[13,14]^ have demonstrated that acupuncture can control the autonomic nerve, and the mechanism of action is mostly represented in the effects of acupuncture on the autonomic nerve at the levels of the cerebral cortex, hypothalamus and pituitary. Heart rate variability (HRV) refers to the small variation between beat-to-beat intervals, which can reflect the relationship between the activity of sympathetic and vagal nerves and the balance and coordination relationship between them. Meanwhile, it can quantitatively reflect the function of autonomic nerve and its effect on cardiovascular regulation.^[9]^ And it is currently the most extensively used indicator to evaluate changes in cardiac autonomic function.^[15]^ Some studies^[16,17]^ found that after acupuncture treatment for stroke patients and patients with autonomic dysfunction secondary to stroke, HRV indexes after treatment were significantly improved compared with those before treatment (*P* < .05). And the degree of improvement was significantly better than the control group (*P* < .05). It is claimed that acupuncture helps treat the autonomic nerve dysfunction induced by stroke.

Therefore, via the objective assessment of HRV detection and clinical feature scale (CFS) ratings, this research further highlighted the immediate impact of manual acupuncture on autonomic nerve dysfunction of varying severity in the convalescence period of stroke. Identify the cumulative impact of the immediate effect and establish that manual acupuncture has various effects on long-term repercussions of severe autonomic dysfunction in the convalescence period of stroke. In order to examine the feasibility and efficacy of acupuncture and moxibustion therapy on autonomic dysfunction in the convalescence period of stroke and to realize its practical value in clinical treatment.

## 2. Methods

### 2.1. Study design

**Figure 1.**
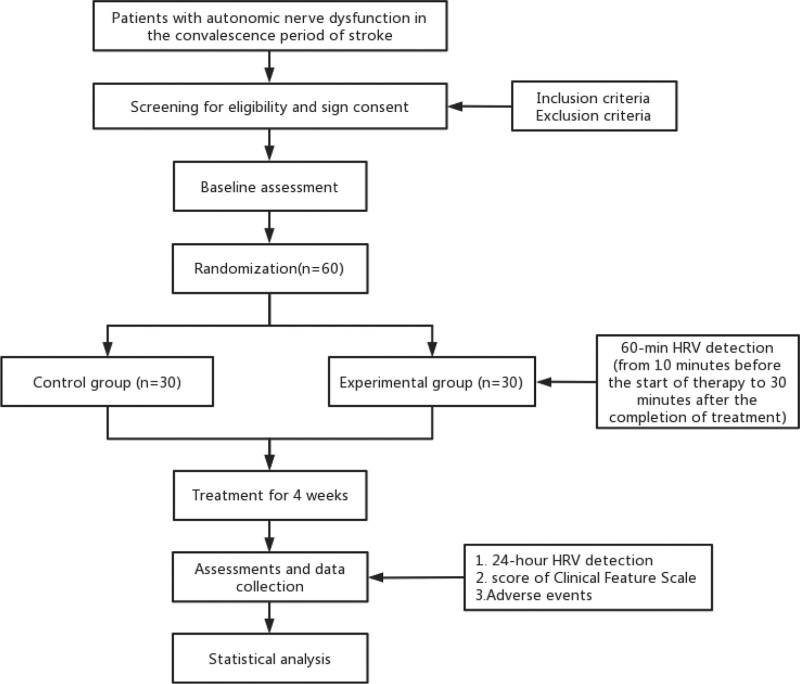
Flow diagram. HRV = heart rate variability.

This randomized, single-blind, controlled clinical trial will be conducted in Longhua Hospital Affiliated to Shanghai University of Traditional Chinese Medicine. A flow diagram of the trial is shown in Figure 1. The schedule of enrollment, interventions and assessments are presented in Table 1.

**Table 1:** The schedule of enrollment, interventions and assessments are presented.

	Sutdy Period					
	Screening	Baseline	Treatment Period (wk)			
Timepoint		*0*	*1*	*2*	*3*	*4*
Enrolment						
Eligibility screen	X					
Informed consent	X					
Allocation		X				
Interventions:						
*Experimental group*			X	X	X	X
*Control group*			X	X	X	X
Assessments:						
*Physical examination*		X				
*Acupuncture Safety and tolerance test*		X				
*Blind method success rate test*		X				
*24-h HRV*		X				X
*60-min HRV*			X	X	X	X
*CFS*		X				X
*Concomitant medication*		X	X	X	X	X
*Adverse reaction record*		X	X	X	X	X

CFS = clinical feature scale, HRV = heart rate variability.

Physical examination include height, weight, blood pressure and heart rate.

### 2.2. Patient recruitment

This study intends to enroll 60 patients who match the diagnostic criteria started in November 2021, all from Longhua Hospital Shanghai University of Traditional Chinese Medicine. The researcher took the initiative to present the study, and the participants volunteered to engage in it. After the doctor completed screening according to the inclusion and exclusion criteria, the participants might be included if they satisfied the requirements. Before randomization, all patients are asked to submit written informed permission.

#### 2.2..1. Inclusion criteria.

1. Meet the diagnostic criteria of Western medicine:
  (1) Patients who meet the diagnostic criteria of cerebrovascular disease adopted by the Fourth National Cerebrovascular Disease Academic Conference in 1995, and confirmed by brain CT and MRI;
  (2) Collect a 24-hour dynamic electrocardiogram from 8 to 9 am on the same day to 8 to 9 am on the next day, and set HRV parameters SDNN < 50ms, SDANN < 40 ms, RMSSD < 15 ms, PNN50 < 0.75%, LF < 300 ms^2^, HF < 200 ms^2^;
2. The first onset is in the 3rd to 6th month;
3. Patients aged 18 to 90 years old;
4. Patients who have signed informed consent.

#### 2.2..2. Exclusion criteria.

1. Pregnant or lactating women;
2. Patients with serious cardiac arrhythmia, myocardial infarction, coronary heart disease, infection, tumor and other serious conditions;
3. Combined with severe liver, kidney, lung, blood and other systemic diseases.

### 2.3. Randomisation and allocation concealment

The subjects will be randomly allocated to an experimental group or control group at a ratio of 1:1 by the random number generated by SPSS V.25.0. The trial was primarily blinded to assess data collectors, data analysts, and outcome judges, and to conceal assessment results and trial results from all investigators, staff, and subjects. The treatment plan adopted by the physician for the subjects should be consistent with the treatment plan corresponding to their random number.

## 3. Interventions

### 3.1. Experimental group

All subjects are able to use basic symptomatic treatment drugs, such as hypotensor, lipid-lowering drugs, hypoglycemic drugs, antiplatelet aggregation drugs, etc.

**Figure 2.**
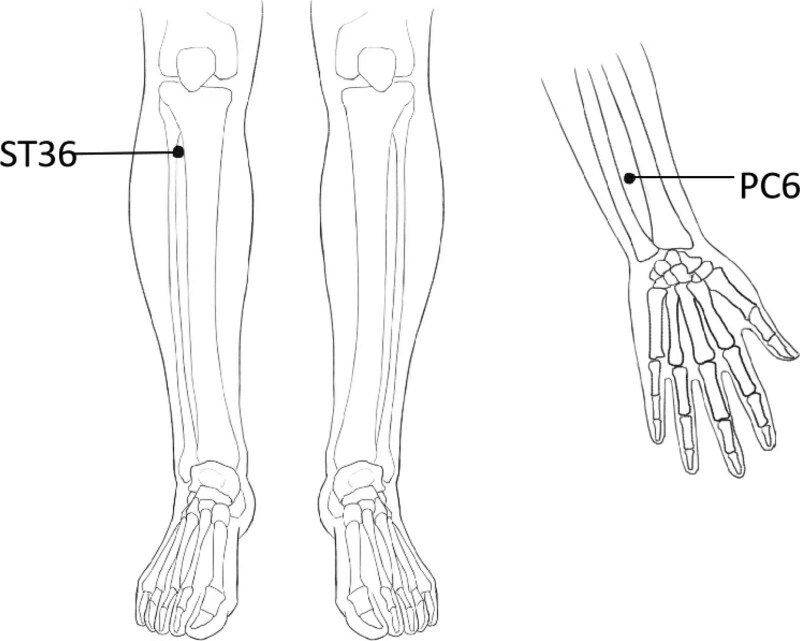
Locations of acupoints. ST36 = Zusanli, PC6 = Neiguan.

On the basis of symptomatic treatment drugs, acupuncture will be performed. The patients in the acupuncture group will lie in the prone position. The selected acupoints are Neiguan (PC6), Zusanli (ST36). All acupoints are localized according to the WHO Standard Acupuncture Locations^[18]^ and are exhibited in Table 2 and Figure 2. After accurate positioning and routinely disinfecting the local skin of the patient and the hands of the physician with 75% ethanol, physician will insert sterile disposable acupuncture needles (Hanyi, 0.25 mm × 40 mm) into the selected acupuncture points and apply the manipulation of even reinforcing and reducing movement for 1 minute, the rotating angle is between 60° to 210°, and the depth is 0.5 to 1 cun (1 cun ≈ 20 mm). The needles will be kept for 20 minutes after stimulating the acupuncture points to reach De qi (a compositional sensation including soreness, numbness, distention, and heaviness), which is believed to be an essential component for acupuncture efficacy. Acupuncture will be performed once every other day, 3 times a week, for a total of 12 times.

**Table 2:** Locations and manipulations of acupoints.

Acupoint	Location	Manipulation
Neiguan (PC6)	Between the palmaris longus tendon and the flexor carpi radialis tendon, 2 cun above the transverse stria of the distal carpometacarpus	Puncture perpendicularly to a depth of 0.5–1 cun with even reinforcing and reducing movement for 1 min
Zusanli (ST36)	Three cun directly below Dubi, and 1 finger- breadth lateral to the anterior border of the tibia	Puncture perpendicularly to a depth of 1–2 cun with even reinforcing and reducing movement for 1 min

### 3.2. Control group

On the basis of symptomatic treatment drugs, symptomatic treatment will be given for the following symptoms of autonomic nerve dysfunction, and clinical observation was conducted for 1 month: If the heart rate is greater than 100 beats/minutes, take propranolol tablets, and the dose is adjusted according to symptoms; if the Ashworth classification of any limb muscle tone improvement is greater than or equal to 3, take clonazepam tablets, and the dose is adjusted according to symptoms; if infection is excluded, body temperature is above 38°C with sweating symptoms, bromocriptine is administered, and the dose is adjusted according to symptoms.

In addition to symptomatic treatment drugs, other related treatments are prohibited during the observation period. Doctors check the patient’s concomitant medication at each visit, and record the name of the drug (or other therapy name), dosage, frequency and time of use in the study medical record “Concomitant Medication Table” for analysis and reporting when summarizing.

## 4. Outcomes

### 4.1. Primary outcome

The primary outcome measure is the HRV detection change, comprising 24-hour HRV detection and 60-minutes HRV detection compared with baseline (0 week) at week 4. 24-hour HRV detection comprises SDNN, SDANN, RMSSD, pNN50%, LF and HF. In addition, patients in the experimental group will be detected for 60 minutes at each time of treatment (from 10 minutes before the start of therapy to 30 minutes after the completion of treatment) to identify the change in LF and HF. The SDNN (the standard deviation of all NN intervals) represents the heart rate variability. SDANN (the standard deviation of the averages of NN intervals in all 5 minutes segments of the entire recording) and LF (power in low frequency range) represent the activity of both the sympathetic nervous system and the parasympathetic nervous system. RMSSD (the square root of the mean of the sum of the squares of differences between adjacent NN intervals), pNN50% (NN50 count divided by the total number of all NN intervals) and HF (power in the high frequency range) represent the activity of the parasympathetic nervous system.

During the HRV test, the patient should be in a supine resting posture to avoid tension, excitement, and deep breathing, and the surrounding environment should be kept calm. The test timing must be between 8 AM to 9 AM on the test day.

### 4.2. Secondary outcomes

The secondary outcome measure is the score change in the Clinical Feature Scale compared with baseline at week 4. With a total score of 0 to 18 points, CFS is used to assess the severity of clinical symptoms of autonomic nerve function. According to heart rate, respiration, blood pressure, body temperature, sweating and posture at the time of seizure, it divides the severity of autonomic nerve dysfunction into 4 grades: normal (0 points), mild (1–6 points), moderate (7–12 points), and severe (≥13 points). All patients will be scored at baseline (week 0) and at the end of treatment (week 4), and the changes in scores before and after treatment will be compared.

### 4.3. Adverse events

Investigators should fill in the “Adverse Event Form” for any adverse reactions that occur during the trial of the subjects, and follow up the investigation, record the treatment process and results in detail, until the laboratory tests return to normal and the symptoms and signs disappear. The investigator decides the diagnosis and treatment measures according to the condition, and decides whether to suspend the observation. If a serious adverse event occurs, the unit undertaking the clinical study must immediately take necessary measures to protect the safety of the subjects. Then the investigator should fill in the “Serious Adverse Event Report Form,” reports it to the Ethics Committee within 24 hours, and signs and dates the report.

### 4.4. Data collection, management and monitoring

The investigator fills in the “Case Report Form” for each subject of this trial. Then it will be reviewed by the clinical monitor and handed over to the data administrator for data entry and management. The case report form can only be underlined when making any evidence-based corrections. The modified data should be signed and dated by the investigator, and the original records should not be rubbed or covered.

### 4.5. Sample size

The study was a case-control study in a group design. Patients recovering from stroke with autonomic dysfunction were tested and compared using the HRV index. According to pre-experiment and literature, HRV index means *δ* = 531.0 and overall standard deviation *s* = 593.7, setting *α* = 0.05 and *β* = 0.1. PASS 11 program calculates *N*1 = *N*2 = 27 for experimental and control cases. We predicted 10% non-response. We plan to recruit 60 participants, 30 in each group.

### 4.6. Statistical analysis

The design of the project was in line with a controlled study. According to the requirements of the standard operating procedures (SOP) for clinical research data management, the statisticians are conducted statistics independently, and are separated from the project research designers and operators. The test was designed by collecting information using the CRF scale. SPSS V.25.0 statistical software (IBM SPSS Statistics) will be used for analysis. The measurement data will be tested for normality and homogeneity of variance. For those with normal distribution were expressed as mean ± SD. The paired t-test will be used for comparison before and after treatment, and the 2 independent samples *t* test will be used for comparison between groups. If the variance is not uniform or does not conform to a normal distribution, the Wilcoxon rank- sum test will be used. The chi-square test will be used for counting data. Unless otherwise stated, hypothesis testing was carried out using 2-sided tests. The test level was *α* = 0.05, with *P* < .05 indicating a significant difference.

## 5. Discussion

Studies have indicated that post-stroke autonomic dysfunction may remain for at least 6 to 9 months,^[6,19,20]^ rather than being a short-term event. Up to today, most randomized controlled studies have noticed the efficacy of acupuncture on patients with autonomic dysfunction in the acute stage of stroke, but few have found the effectiveness of acupuncture on autonomic dysfunction throughout the convalescent phase after stroke. Therefore, it is worth additional investigation on the effectiveness of acupuncture for improving autonomic dysfunction in the convalescence period of stroke.

Studies have revealed that acupuncture has a bidirectional adjustment to the heart rate of healthy people, which is equivalent to the impact of acupuncture on balancing yin and yang,^[21]^ and the vagus nerve is the key reaction.^[22,23]^ Modern studies^[23–26]^ have found that Neiguan is close to the median nerve and overlaps with the innervated nerve segments of the heart, and the information of acupuncture at Neiguan can reach the cardiovascular center, namely the nucleus of the solitary tract, the posterior hypothalamus and the preoptic area-the front part of the hypothalamus. In the early stage of our research group, a patient with mild autonomic dysfunction in the convalescence period of stroke and a severe patient were acupunctured with bilateral Zusanli and bilateral Neiguan, respectively, and we found that the HRV values of the 2 patients reached equilibrium at 20 minutes and 25 minutes of acupuncture. Based on prior study findings, we anticipated that acupuncture at Neiguan and Zusanli might ameliorate autonomic nerve dysfunction after stroke. Patients exhibied immediate effects with varying durations following acupuncture for 20 minutes, and the initial effects might be cumulative to develop long-term benefits. Studies^[27]^ have shown that acupuncture at Zusanli can activate parasympathetic nerve activity and inhibit sympathetic nerve activity, and Zusanli is an essential acupoint for health care. Its anti-inflammatory effects,^[28]^ antioxidant effects,^[29]^ and effects in enhancing gastrointestinal immunity,^[30]^ regulating intestinal flora and brain-gut peptides,^[31]^ and stabilizing the intestinal micro-ecological environment^[32]^ have been widely proved. Therefore, Neiguan and Zusanli were chosen for this investigation.

There are still some limitations in this study. In the first place, the study aim is patients in the convalescence period of stroke, but there is no clear differentiation between whether stroke is caused by cerebral hemorrhage or cerebral infarction. In another, the sample size is not large enough.

However, this experiment satisfied the methodological standards of randomization, single-blindness, control, outcome assessors, and statisticians. In this work, the objective assessment approach of HRV detection along with the CFS scale was employed to measure the development of autonomic dysfunction throughout the convalescence phase after stroke. The outcomes of this pilot trial will offer a high-quality foundation for examining the effectiveness and safety of acupuncture in the treatment of autonomic dysfunction in the convalescence stage after stroke, and will open up a new field of clinical acupuncture treatment.

## Author contributions

**Conceptualization:** Shijing Jia, Wang Lu.

**Data curation:** Yijin Guo, Jiabao Zhang, Xinyu Zhang, Yimeng Wang.

**Funding acquisition:** Wang Lu.

**Investigation:** Chu Zhang, Yunqi Lu, Shenghong Zhang.

**Methodology:** Kun Xue.

**Writing – original draft:** Shijing Jia.

**Writing – review & editing:** Wang Lu, Minghui Hang, Zilin Ma, Haiyin Zhao.
